# Mycorrhizal Patterns in the Roots of Dominant *Festuca rubra* in a High-Natural-Value Grassland

**DOI:** 10.3390/plants11010112

**Published:** 2021-12-30

**Authors:** Larisa Corcoz, Florin Păcurar, Victoria Pop-Moldovan, Ioana Vaida, Vlad Stoian, Roxana Vidican

**Affiliations:** 1 Department of Microbiology, Faculty of Agriculture, University of Agricultural Sciences and Veterinary Medicine Cluj-Napoca, Calea Mănăştur 3-5, 400372 Cluj-Napoca, Romania; larisa.corcoz@usamvcluj.ro (L.C.); victoria.pop@usamvcluj.ro (V.P.-M.); roxana.vidican@usamvcluj.ro (R.V.); 2 Department of Grasslands and Forage Crops, Faculty of Agriculture, University of Agricultural Sciences and Veterinary Medicine Cluj-Napoca, Calea Mănăştur 3-5, 400372 Cluj-Napoca, Romania; ioana.vaida@usamvcluj.ro

**Keywords:** plant–fungal interaction, grassland ecosystem, symbiosis, mycorrhizal maps, fungal strategy, MycoPatt, colonization patterns

## Abstract

Grassland ecosystems occupy significant areas worldwide and represent a reservoir for biodiversity. These areas are characterized by oligotrophic conditions that stimulate mycorrhizal symbiotic partnerships to meet nutritional requirements. In this study, we selected *Festuca rubra* for its dominance in the studied mountain grassland, based on the fact that grasses more easily accept a symbiotic partner. Quantification of the entire symbiosis process, both the degree of colonization and the presence of a fungal structure, was performed using the root mycorrhizal pattern method. Analysis of data normality indicated colonization frequency as the best parameter for assessing the entire mycorrhizal mechanism, with five equal levels, each of 20%. Most of the root samples showed an intensity of colonization between 0 and 20% and a maximum of arbuscules of about 5%. The colonization degree had an average value of 35%, which indicated a medium permissiveness of roots for mycorrhizal partners. Based on frequency regression models, the intensity of colonization presented high fluctuations at 50% frequency, while the arbuscule development potential was set to a maximum of 5% in mycorrhized areas. Arbuscules were limited due to the unbalanced and unequal root development and their colonizing hyphal networks. The general regression model indicated that only 20% of intra-radicular hyphae have the potential to form arbuscules. The colonization patterns of dominant species in mountain grasslands represent a necessary step for improved understanding of the symbiont strategies that sustain the stability and persistence of these species.

## 1. Introduction

Grasslands are one of the most widespread types of ecosystems in the world, characterized by the presence of herbaceous species that are grown close to the ground because of their main use as animal feed [1]. Unfavorable environmental conditions (e.g., lack of nutrients, poor water regime, soil pH) and improper grassland management represent a constant pressure on the stability of grasslands [2]. These ecosystems occur naturally on all continents, with an estimated area of 52.5 million km^2^, or 40.5% of the earth’s surface, except Greenland and Antarctica [3]. Their main function is productivity, but they additionally control solar energy, produce complex organic matter, are an important source of food for animals, and have a high ecological value for the diversity of fauna, which, in turn, supports human livelihoods [4].

Generally, the functionality of all ecosystems is determined by the biodiversity and assemblage of the species present. Grasslands are characterized by high diversity and are the main key to maintaining the flora, fauna, and human populations worldwide [5]. A special case is the alpine grasslands, considered to have high natural value (HNV), which offer multiple ecosystem services, such as fodder production, and are especially a biodiversity reservoir. Grasslands are also important carbon sinks, as well as important sites for greenhouse gas reduction, water regime regulation, and biodiversity protection and conservation [6,7]. Current climate changes with the excessive use of fertilizers and pesticides [8] to increase yields amplify the pressure on grasslands. To maintain the functionality of these ecosystems, harmonious management is necessary to sustain their climate. Due to agricultural intensification, more arable land is needed, and the grasslands were the first to be converted [9,10]. In addition to the conversion of grasslands into arable land, excessive and irrational grazing have been practiced in the recent decades. Both processes have devastating effects, resulting in species extinction and favoring the establishment of invasive species. Human activities are the main driving force in terrestrial ecosystems [11]. Abiotic stresses are responsible for more than half of grassland depreciation worldwide [12,13]. In this context, grassland protection has been a major goal lately in order to ensure their food security and sustainability.

The soil that supports grasslands is the most dynamic and favorable habitat for soil microorganisms [14]. These microorganisms regulate the establishment and proper development of terrestrial species as well as the elementary processes underground, such as decomposition of organic matter [15], nutrient flow, and maintenance of soil structures [16]. The dynamic of the vegetation assemblage is directly correlated with the below-ground dynamic, especially because both the microbiota and edaphic microorganisms are adapted to oligotrophic conditions [17]. Grasslands have the greatest diversity of biomes at the terrestrial level, being proportional to the high number of plants, animals, and microorganisms and the full flux of relationships they develop in the ecological niche occupation [18]. Soil microorganisms, especially fungi and bacteria, provide stability through the scale of the processes that shape the structure of the soil and its evolution [19]. Microflora performs a wide range of activities, especially the decomposition of organic matter, the release and transformation of nutrients to plants, and the degradation of toxic residues [20]. This is visible in a complex mechanism where plant phylogeny and species identity coordinate microbiota patterns in the rhizosphere [21].

One of the most important symbiotic relationships present in grasslands is between plant roots and fungi (mycorrhizae). This relationship has been recognized since the early 19th century, and fungi are the most widespread in the world. Mycorrhizal associations are ubiquitous in ecosystems and in species of interest from among agricultural crops, grasslands, and forests. Approximately 40,000–50,000 fungal species form mycorrhizal associations with nearly 250,000 plant species [22]. These associations vary in structure and function, but the most common interactions are of the arbuscular type. It is estimated that the percentage of terrestrial plants that establish this type of association exceeds 80% [23], from angiosperms to gymnosperms. Arbuscular mycorrhizal fungi link the functioning of the ecosystem above and below ground and, through their contribution to the nutrient cycles and plant productivity, are among the most important organisms in the soil [24]. Natural grasslands are characterized by slower nutrient cycles, which are related to the specific requirements of plant communities [25]. In addition, the competition between different taxa in these ecosystems enhances the formation of microbial–plant partnerships, within which mycorrhizas act as regulators. These mechanisms are visible in the selection of rhizospheric microbiomes, and each plant represents a specific niche for microorganisms [26].

According to the literature, this symbiosis between vascular plants and fungi can be expressed as a percentage in terms of mycorrhizal fungus taxa as follows: 72% plants form symbioses with arbuscular mycorrhizal fungi, 10% represent symbioses with mycorrhizal fungi of the orchidaceous type, 8% represent species that do not form mycorrhizal-type symbiosis, 7% have inconsistent mycorrhizal or non-mycorrhizal symbiosis, 2% have symbiosis with ectomycorrhizal fungi, and 1.5% establish symbiosis with mycorrhizal fungi of the ericoid type [27]. All types of mycorrhizae can be found in grassland ecosystems. Grasses occupy the largest area in practical ecosystems, this being correlated with the degree of connection to the mycorrhizal symbiosis of most species. Helgason et al. [28] proved that mycorrhizal symbioses can balance the competitive relationship between native and invasive species through different strategies provided by the fungi as symbiotic partners in the roots. The mycorrhizal community is one of the key factors for increasing the competitiveness of plant communities and determining the structure and stability of plant species [29]. By understanding the native process of mycorrhizal colonization in the roots of dominant species, the biological mechanism for maintaining the plant community stability can be clarified and even future successions can be predicted.

Among the dominant species in Romanian grasslands, the grass species *Festuca rubra* is an exponent of adaptability to a wide variety of site conditions, especially in ecosystems managed by low inputs [30]. Environmental conditions, especially the low reserve of nutrients in the soil, have created an evolutionary state for the association of this species with mycorrhizal fungi in order to obtain the resources necessary for optimal growth and development [31].

The need for both visual and mathematical results from the analysis of mycorrhizal abundance and function is vital for understanding plant communities’ assemblages, especially in perennial plants found in grasslands. Studies have provided valuable information regarding the symbiotic basis of plant survival and success in competition with other taxa. The MycoPatt tool [32] analyzes the mycorrhizal extension patterns in roots and extracts three forms of all the colonization parameters. The capacity of this tool for comprehensive analysis of mycorrhized roots makes it useful for the detailed assessment of colonization, even for arbuscule development. This structure can be traced during each step of arbuscule formation, with a deeper analysis of root reactions that can lead to arbuscule suppression, their dimension in root cells, and even their dissolution. All root samples are observed under a microscope, and all images from each segment are extracted as the primary database. Next, a grid is applied over the images, and the identification of each structure is developed by fungal symbionts. During this process, each structure is coded by a number: one for hyphae, two for arbuscules, three for vesicles, four for spores, five for auxiliary cells, and six for entry points. All these numbers are input into the MycoPatt tool, which converts them into colors and calculates the colonization parameters: the frequency and intensity of colonization (%), the presence of arbuscules and vesicles (%), the colonization degree (%) as a volumetric expression of mycorrhizal extension, the percentage of non-mycorrhized areas, and the report between mycorrhized and non-mycorrhized areas. The colonization maps exported by this tool show colonization parameters in graphical form, which present the real position of each structure in the roots and result in increased objectivity of mycorrhizal assessment.

The main aim of this paper is to use the MycoPatt tool to assess the native mycorrhizal potential of *Festuca rubra* in a long-term mowed mountain grassland ecosystem. The tested hypotheses fall in the methodology sphere regarding mycorrhizal mechanism analysis and data analysis of the mycorrhizal functionality. The following hypotheses were proposed:

(i). Is the colonization frequency a parameter for defining the primary permissiveness of the root toward the symbiont?
(ii). Does the intensity of colonization define the secondary permissiveness of the root toward the symbiont?
(iii). Is the intensity of colonization dependent on frequency?
(iv). Is mycorrhizal colonization a stable mechanism, or does it have fluctuations in the root?
(v). Is the mycorrhizal pattern linear or fluctuating, and how sensitive is the change?
(vi). Is the complete exploration of colonization data and maps efficient in identifying colonization strategies?
(vii). Is the assessment of the colonization dynamics by the MycoPatt tool effective in the real positioning of the symbiont in the root cortex?

Our results represent a good comparison database for future research on mycorrhizas in grasslands, with numerous patterns that show a higher potential of colonization strategies. Each structure was assessed based on its real position in the host roots, and the entire colonization process was objectively described. The results also represent the proposal of a new methodological frame to better analyze the mycorrhizal colonization process and to organize the obtained results.

## 2. Results

### 2.1. Establishment of Best-Class Solution for Grouping Mycorrhizal Parameters

The MycoPatt tool is extremely useful because of the large amount of data obtained and the increased potential to exemplify the biological mechanisms related to mycorrhizal colonization. The database used for this projection contains 900 observations, which represents the potential of the entire symbiotic process between soil-native mycorrhizas and *Festuca rubra*. Due to the large amount of data, it is necessary to classify mycorrhizal parameters for further processing in order to improve the observation resolution.

**Figure 1:**
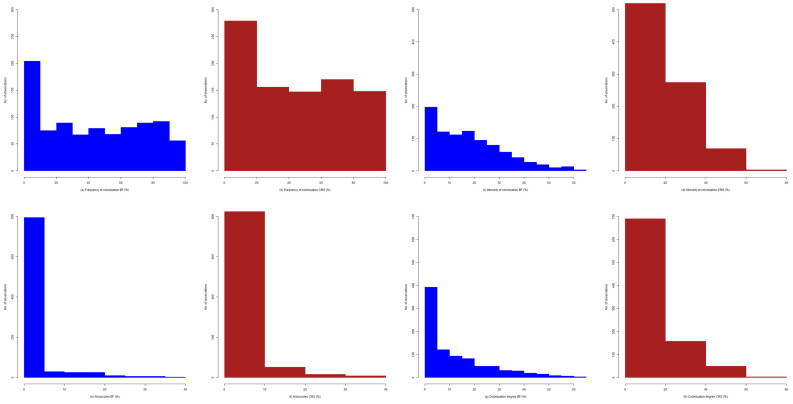

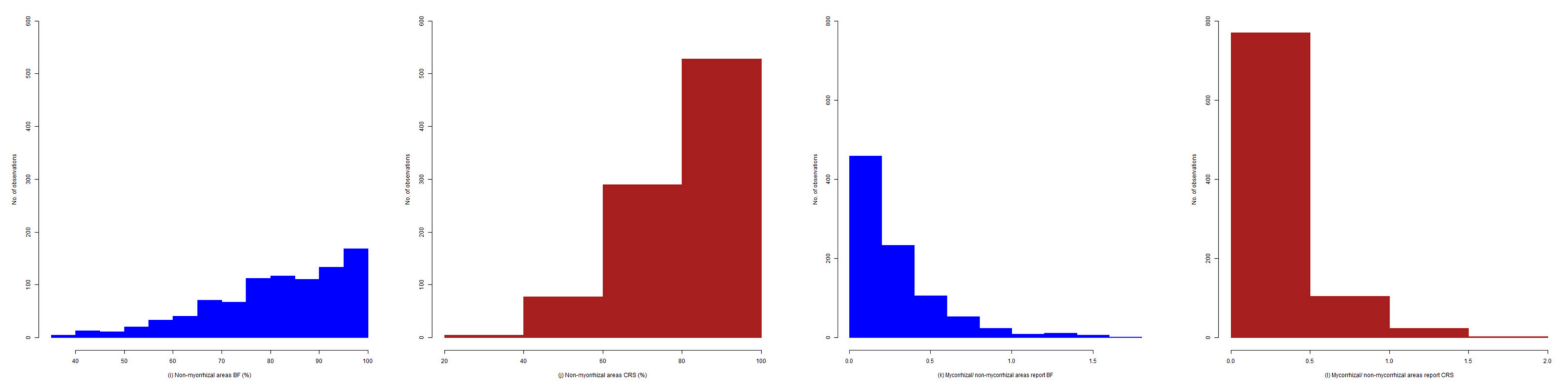
Histograms of colonization parameters. Each parameter was analyzed through two solutions: BF, best-fitted solution, given by the basic histogram formulas; CRS: frequency-class-restricted solution, given by the restriction of the frequency classes M1, low level (0–20%); M2, low–medium level (21–40%); M3, medium level (41–60%); M4, medium—high level (61–80%); and M5, high level (81–100%). (**a**) Frequency of colonization BF (%), (**b**) Frequency of colonization CRS (%), (**c**) Intensity of colonization BF (%), (**d**) Intensity of colonization CRS (%), (**e**) Arbuscules BF (%), (**f**) Arbuscules CRS (%), (**g**) Colonization degree BF (%), (**h**) Colonization degree CRS (%), (**i**) Non-mycorrhizal areas BF (%), (**j**) Non-mycorrhizal areas CRS (%), (**k**) Mycorrhizal/non-mycorrhizal areas report BF, (**l**) Mycorrhizal/non-mycorrhizal areas report CRS.

The first step in data assessment was testing the normality to highlight the level reached by each of the intra-radicular colonization parameters. In this sense, the frequency histograms were elaborated based on the optimal solution presented by the statistical test (Figure 1). The advantage of the MycoPatt tool is the general report at the 100% level and the permissiveness of a unitary exploration between 1 and 10%. Due to the large number of observations, the frequency histograms were also created by hierarchizing the data into five classes. This option was chosen because of the chaotic data distribution from the first histograms. The frequency parameter ranged between 0 and 100% and was represented by the presence of any structure of the fungal component in the root cortex. Therefore, the data set was divided according to this parameter to test the general trend of the symbiont process within *Festuca rubra* in the grassland ecosystem. Five classes were used for the histogram design in the following ranges: M1 from 0 to 20%, low colonization level; M2 from 21% to 40%, medium–low colonization level; M3 from 41% to 60%, medium colonization level; M4 from 61% to 80%, medium–high colonization level; and M5 from 81% to 100%, high colonization level. These five classes highlighted the trends’ appearance and narrowed the ranges within the colonization parameters.

Based on the data obtained from research in terms of the parameter frequency of root colonization in *Festuca rubra,* the data distribution was not uniform; most data (over 200) were found in the 0–10% range (Figure 1a). At 10–20%, 30–40%, and 50–60%, the data distribution was similar, being recorded from the same number of observations. In these three ranges, there were about 70 observations. The other ranges, except for 90–100%, had a higher number of observations compared with the previous ones. The range with the fewest observations was given by the last ranking place, which presented values between 90% and 100%, here being recorded slightly lower around 50 observations. The observations in this range could be explained by the fact that the symbiotic partner rarely reaches frequency parameter values of over 90%. An interesting aspect was shown by the 60–90% range: the observations were in ascending order. An improvement in graphic projection was obtained from the frequency histogram classified into the defined five classes (Figure 1b). A more uniform data distribution could then be observed; however, the number of observations at different ranges was maintained. The range with the highest recorded data was the first (0–20%), with approximately 300 data points found. The second rank was given to the 60–80% range. The 20–40% range had a slightly lower number of observations than the previous range. A similar observation set was obtained for the two ranking positions of 40–60% and 80–100%. 

The histogram with the best-fitted solution for the root colonization degree assessing *Festuca rubra* (Figure 1c) followed a downward slope, with the maximum number of data points and intensity value between 0 and 5%, with over 200 observations recorded. Due to the lower values of this parameter, the histogram consisted of 13 short-range intervals. From the 20% range, the number of data decreased with increasing intensity parameter value. The fewest observations were found in the range in which the intensity value increased above the 60% threshold. For the histogram in which the data were grouped into five descending classes, the trends were maintained, with four ranges for the intensity values (Figure 1d). Most data sets were in the 0–20% range. The range comprised about 66% of the total number of observations. The second range that included an impressive number of observations was the 20–40% range, which comprised about 33% of the total number of observations. A few observations were found in the 40–80% range, which can be attributed to the cell rigidity in the root cortex. This indicated that the autotrophic partner accepts the symbiont but without intimate contact between them. Specifically, it allows it to develop intracellularly but without forming specific structures or allowing intracellular development.

The structures specific to the fungal component provided a representative arbuscule histogram (Figure 1e,f). These structures represented the bilateral acceptance of the symbiosis process, with their help in nutrient transfer. The values of this parameter did not exceed 40%, and the observations were divided into eight intervals. Most observations pointed out transfer structures with values of a maximum 5%, resulting in about 800 observations. The 5% and 20% ranges consisted of the same data sets, and the 20–40% range showed few observations. This indicates that *Festuca rubra* has high rigidity for the fungal component, more precisely for the involvement in the entire symbiont process. The histograms formed based on the five chosen classes were similar, presenting four intervals as well, and the maximum value of the arbuscules did not exceed 40%. In the 0–10% range, over 93% of the total number of observations were found. From values above 10%, a downward slope was observed, indicating the inability of the fungal component to form numerous structures with a role in nutrient transfer.

The root volume colonized by mycorrhizal fungi, expressed by the colonization degree, showed a downward trend proportional to variations in frequency and intensity (Figure 1g). The set of observations based on the colonization degree parameter was divided into 13 intervals. Most data sets were found in the 0–5% range, with this being present in about half of the entire data set. An interesting aspect was seen in the 20–30% range, which uniformly presented the same data sets. The colonization degree over 50% showed a significant reduction in the symbiotic process. The grouping of the data into the five classes maintained the downward trend of the histogram for the colonization degree (Figure 1h). The peak of the colonization number was found in the 0–20% range, with about 95% of the total number of observations being present. The 20–40% range had a high number of data points, and above values of more than 60%, few mycorrhizal structures were found.

The perspective of non-colonized areas indicated numerous areas of the root without the presence of the fungal component (Figure 1i). Most observations were in the 75–100% range. This phenomenon is mainly due to the plant’s perennity, which involves a senescent root system with low annual growth and also the maintenance of the hyphae system to support nutritional requirements. The histogram of the five classes showed the data set as an ascending slope (Figure 1j). In the 20–40% range, there was a low number of data points. The 40–60% range had about 80 observations. The 60–80% range comprised 300 observations, and the 80–100% range had the most data sets with over 500 observations.

The mycorrhizal/non-mycorrhizal area ratio was generally 0.0–0.5%. This aspect indicates low permissiveness of the root system to abundant colonization and for entry points of new hyphae (Figure 1k,l).

### 2.2. Dynamics of Colonization Parameters

The second step in the analysis of mycorrhizal colonization in *Festuca rubra* was the assessment of the differences due to the five classes at the level of the grassland ecosystem (Table 1). In terms of frequency, the variation range was between 6 and 93%. The differences between classes were significant (*F* = 22,748, p < 0.001). The maximum frequency value was reached in the M5 class. Afterward, it decreased by about 20% per class. With regard to intensity, the variation range was from 3 to about 40%. The differences between classes were significant (*F* = 3103, p *<* 0.001).

The highest intensity value was found in the M5 class, indicating that high frequency also induces high intensity (Table 1). The difference between M5 and M4 classes in terms of intensity was about 10%, and subsequent classes showed a gradual decrease of 7%. The lowest intensity value was present in the M1 class, indicating that the low frequency of the symbiotic process induces a low intensity. The values observed for arbuscularity were between 0.14 and 5%. The differences between classes were significant (*F =* 77.97, *p* < 0.001). The maximum value was recorded in the M5 class, indicating that at high frequency and intensity, the fungal component is able to develop these transfer structures. The differences between M5 and the other classes, as well as between M4 and M1 classes, were significant. The colonization degree was between 0.42 and 36%. In addition, the differences between classes were significant (*F* = 3038, p *<* 0.001). The frequency was correlated with the degree of colonization because the frequency values were previously filtered in ascending order, with significant differences between classes. This result suggests that at low frequency, the colonization degree decreases, and that increased frequency generates a high colonization degree. The impact of frequency was higher than the intensity in this synthetic index. For non-mycorrhizal areas, the variation range was between 61 and 96%. Significant differences between the analyzed classes (*F* = 3103, p *<* 0.001) followed an inverse downward trend proportional to the colonization degree. Regarding the mycorrhizal/non-mycorrhizal area ratio, the variation range was between 0.04 and 0.7%. The values showed significant differences (*F* = 1433, p *<* 0.001), with an upward trend of 10 units in the frequency range of 0–60% and 15 and 27 units in the frequency range of 60–100%.

### 2.3. Analysis and Forecast of the Colonization Development in the Root Cortex

**Figure 2:**
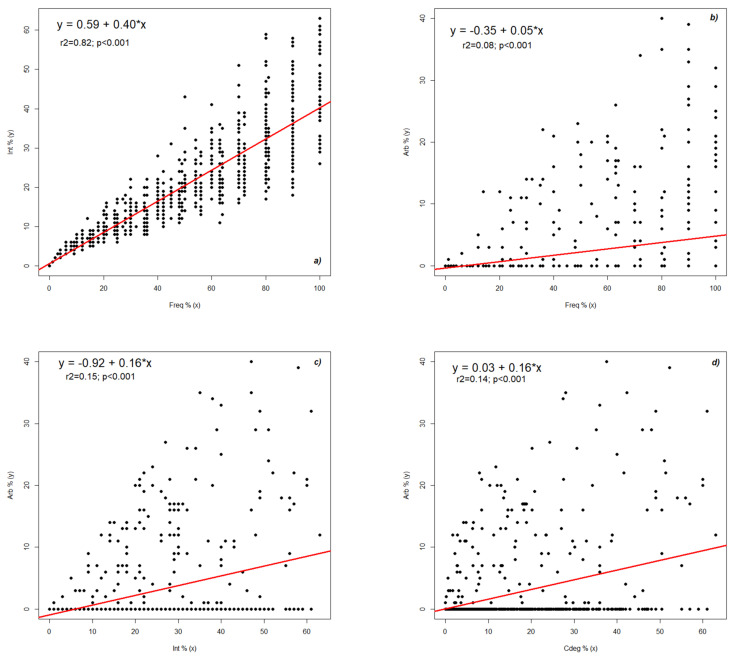

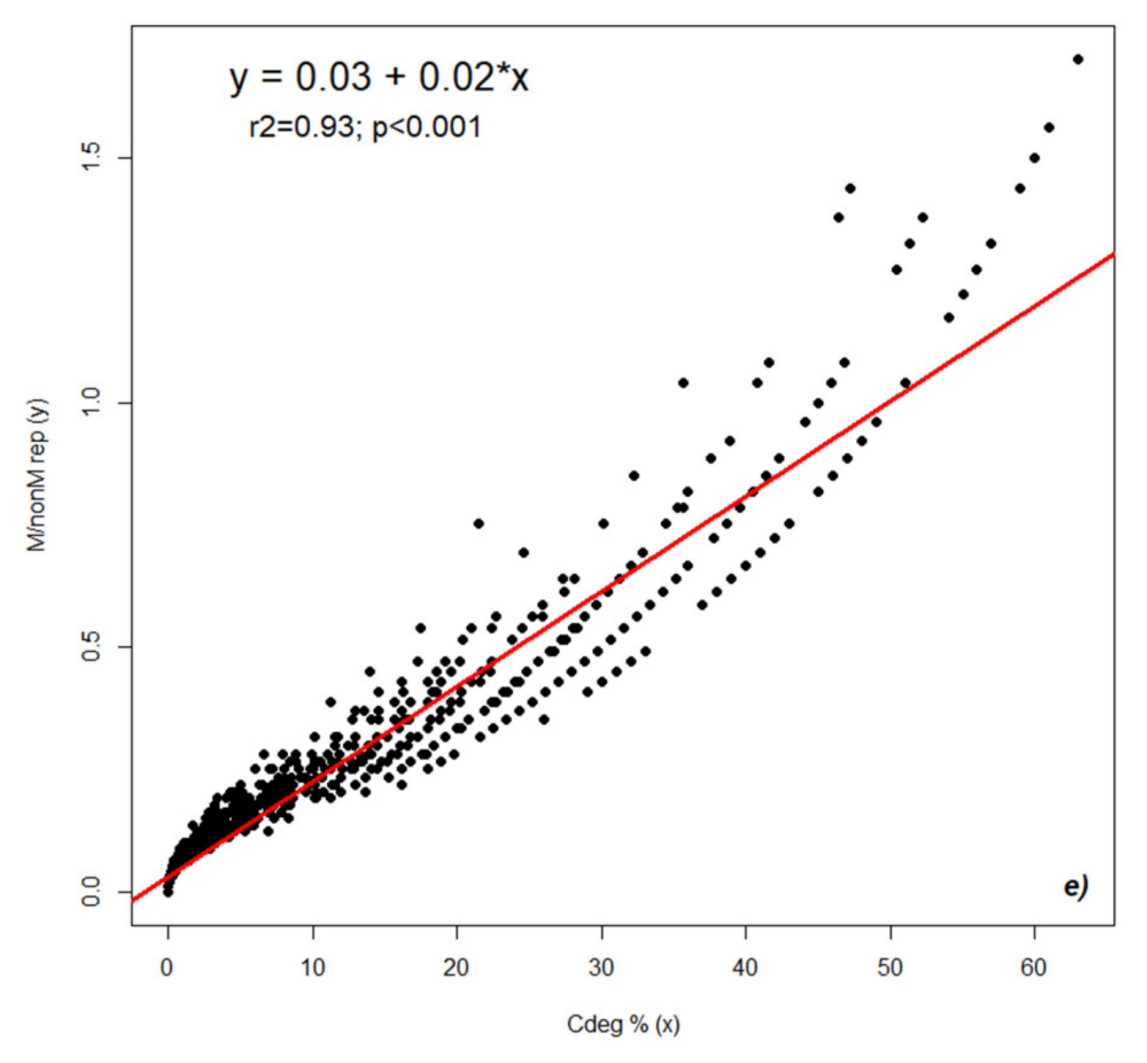
The interdependence of mycorrhizal parameters in the roots of *Festuca rubra*: (**a**) frequency vs. intensity, (**b**) frequency vs. arbuscules, (**c**) intensity vs. arbuscules, (**d**) degree of colonization vs. arbuscules, and (**e**) degree of colonization vs. mycorrhizal/non-mycorrhizal area ratio. Legend: Freq—frequency of colonization; Int—intensity of colonization; Arb—arbuscules abundance; Cdeg—colonization degree; M/nonM rep—mycorrhizal/non-mycorrhizal area report.

The high value of the correlation between frequency and colonization degree led to an additional evaluation of the data distribution according to both parameters (Figure 2a). Following the first analysis, a balanced increase in both parameters was observed up to a frequency value of 20% and 10% of intensity above this level. The deviation from normal was 10–30% in the case of intensity and only 5% in the case of frequency. This observation indicates a constant root penetration but a large fluctuation in the success of colonization. Once hyphae enter the roots, they are not always able to develop abundantly. The highest intensity variations were observed at a frequency of over 50%, when the intensity decreased. In the 90–100% range, the intensity increased. The regression equation sets the base level of the colonization degree below 1%, supplemented by 0.4 units for each additional frequency percentage. There were also some observations that deviated greatly from the interval of which they were a part, which can be explained by all the factors that contribute to the development of this symbiotic process. At a frequency value of 100%, the intensity value should not exceed 40% (Figure 2a).

To better understand the relationship between arbuscules and frequency, it was essential to analyze their tendencies (Figure 2b). Following a preliminary analysis, an almost linear distribution of frequency was observed up to a value of 15%, after which the data were chaotically distributed. At the maximum frequency, the value of the arbuscules should not exceed about 5%. Because the graph shows values of arbuscules up to 40%, it was not possible to generalize the influence of frequency on the arbuscules and the oscillation of values was strong. The regression equation sets the base level of the arbuscules at below 0.5%, supplemented by 0.05% units at each additional percentage of frequency. The chaotic data distribution could be attributed to a biological process that showed fluctuations, and we could not say that the fungal component developed in the cells of the root cortex always manages to form its specific structures.

The graphic pattern also showed fluctuations for the relationship between the intensity of root colonization and arbuscules (Figure 2c). Most of the registered data were distributed at higher values than normal. At the maximum intensity, the number of arbuscules should not exceed 5%. However, in the graph, we found values for this parameter even at 40%. The regression equation sets the base level of the arbuscules at less than 1%, supplemented by 0.16 units for each additional intensity percentage. Since we found a more uniform distribution in this graph from recorded normal values, the intensity of root colonization can be said to more influence the formation of arbuscules than the frequency of the entire process. The model in the graph supports three possible approaches to the colonization mechanism. The area at the base of the graph indicates the lack of intracellular penetration and the formation of arbuscules. In this case, the strategy of the fungus is to strongly colonize the root without developing intimate contact with the cortical cells. The second mechanism is based on the general regression model, which establishes a maximum potential of arbuscules below 10% at a colonization degree of 50–60%. This suggests that only 20% of intraradicular hyphae can develop arbuscules. A third mechanism is visible in the maximum area of the registered arbuscules, stabilizing the production potential at 40% of the intraradicular hyphae. This mechanism establishes the maximum potential for contact between the two partners.

The relationship between the root colonization degree and the transfer structures was similar to the intensity, with the values of the arbuscules being slightly lower (Figure 2d). The regression equation sets the base level of the arbuscules at below 0.05%, supplemented by 0.16 units for each additional colonization degree percentage. An interesting aspect was the linear distribution of normal data and the presence of most data up to a value of 30% colonization degree.

The colonization degree was represented by the presence of the fungal component and also by its specific structures in the root cortex of the autotrophic species (Figure 2e). The relationship between this parameter and the mycorrhizal/non-mycorrhizal area ratio is presented in Figure 2e. The colonization degree oscillated up to 60%, and the mycorrhizal/non-mycorrhizal area ratio went up to about 2. The regression equation sets the basic level of the mycorrhizal/non-mycorrhizal area ratio at less than 0.05%, supplemented by 0.02 units for each additional colonization degree percentage. As can be seen from the graph, at 50% of the colonization degree, the maximum value of the ratio should not exceed 1%.

### 2.4. Exploratory Analysis of Root Colonization in Festuca rubra

**Figure 3:**
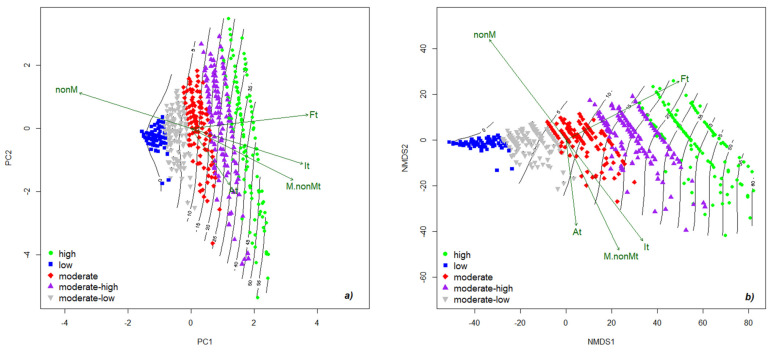
PCA (**a**) and NMDS (**b**) projection of the mycorrhizal pattern and their gradient in the roots of *Festuca rubra*. The five classes are based on the frequency of colonization classes: M1, low level (0–20%); M2, moderate–low level (21–40%); M3, moderate level (41–60%); M4, moderate–high level (61–80%); and M5, high level (81–100%). Legend: Ft—frequency of colonization; It—intensity of colonization; Arb—arbuscules abundance; nonM—non-mycorrhizal areas; M.nonMt—mycorrhizal/non-mycorrhizal area report.

PCA was conducted to determine the relationship between the frequency of root colonization and the other parameters of root system colonization in *Festuca rubra* (Figure 3a). PCA allowed the exploration of both observations of mycorrhizal colonization and the projection of colonization parameters relative to the synthetic colonization degree index. In addition, the dispersion of the values recorded for each factor studied and the spatial projection of the vectors relative to each parameter of colonization were recorded. The quality of microscopic observations was supported by antagonistic gradients of intensity and non-colonized areas, with a spatial orientation in the ++ and −− quadrants, respectively. The total variance explained by PCA was 98.25 (axis 1: 94.25%; axis 2: 3.89%). Up to a colonization degree of 5%, the first two classes were found: those that had low and medium–low frequency. For these two classes, the observation tendency was toward non-colonized areas, to the detriment of the acceptance of the fungal partner. For the low-frequency class, the data were almost homogeneous, with only two data points more distant. In the case of the medium–low frequency class, the colonization degree reached the quadrant at 10%. Only one data set was present at the colonization degree over 10%. The data were evenly distributed in the ++ quadrant for the medium-frequency class and were represented a little more chaotically in the −− quadrant. The + quadrant was crossed by the frequency vector, and the quadrant highlighted the presence of the intensity vector, the arbuscules, and the mycorrhizal/non-mycorrhizal area ratio. Because the data were represented more chaotically in this quadrant, we can hypothesize that the intensity value is strictly correlated with the presence of transfer structures and induces more instability in the symbiotic process than the frequency vector. For the medium–high frequency class, the colonization degree oscillated between 15% and 45%. The data in the + quadrant were represented linearly and homogeneously, whereas the data in the quadrant were more scattered and oscillating. For the high-frequency class, the colonization degree oscillated between 30% and 60%. Most of the data were found in the ++ quadrant, which may indicate that high values of colonization degree are determined by high values of frequency. The data in the −− quadrant showed higher instability due to the intensity values that stimulate the conversion of hyphae into transfer structures.

Non-metric multidimensional scaling (NMDS) analysis was performed to demonstrate the effect of frequency on other parameters of the fungal community in *Festuca rubra* (Figure 3b). The ordination was similar to the one from PCA; however, in the NMDS graph, the relationship between the parameters could be observed in detail. The lengths of the vectors that are part of the graph were maintained, as was the relationship between them. Up to a colonization degree of 5%, there were classes with low and medium–low frequency. In these two classes, the dominant vector was given by the absence of the fungal component. At values of up to 25%, most data showed a high frequency, and above this value, most data tended to show a high colonization degree with numerous arbuscules as nutrient transfer structures.

### 2.5. Colonization Strategy in Festuca rubra

**Figure 4:**
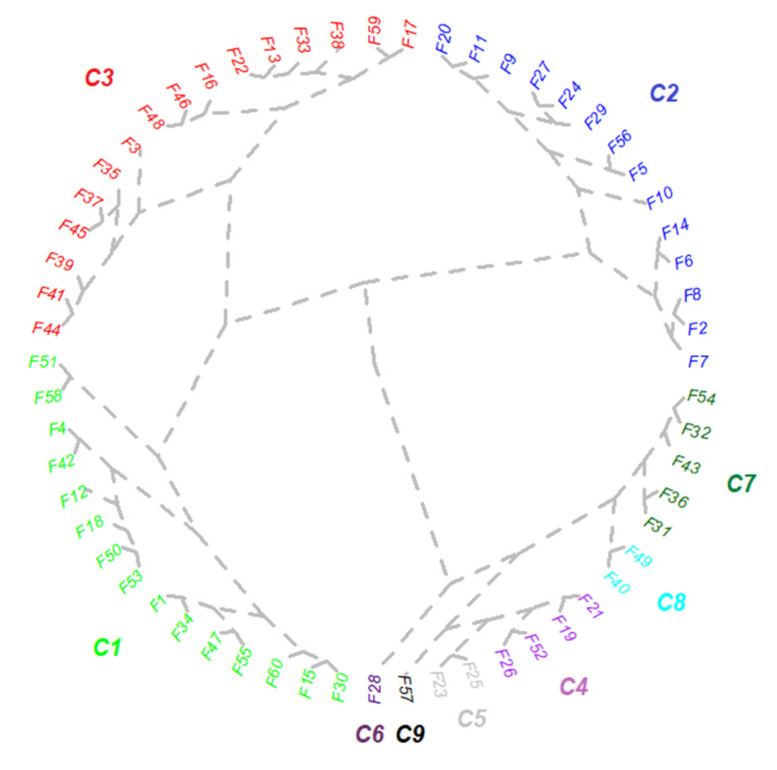
Cluster analysis of the mycorrhizal strategy based on the colonization mechanism in the roots of *Festuca rubra*. Each cluster is defined by the similarity between colonization parameters, with the best solution resulting in nine clusters. F followed by a number, *Festuca rubra* and the segment number (1–60). The conceptual hypothesis is that each segment may represent a specific colonization pattern, and based on linkages, each cluster defines a colonization strategy with similar colonization patterns (detailed in Table 2).

Cluster analysis provides numerous observations on the relationship between colonization parameters and the strategy of colonization in the root system. Based on the recorded data, a new database was created according to the average value of each class. The obtained dendrogram was divided into nine clusters (Figure 4, Table 2). The first cluster (C1) comprised 25% of all the data and represented the second-largest cluster in the entire dendrogram. The data in this cluster showed a 41% colonization frequency, lower than the average, and the intensity was set to 14%. These observations did not record arbuscules. The degree of colonization was 9%, and in accordance with this value, the non-memorized areas occupied a significant area.

Approximately 23% of all data came from C2, and it was ranked third in size in the entire dendrogram (Figure 4, Table 2). The observations that constituted this cluster had the lowest frequency of about 19%. The intensity was half of the frequency for these observations. The arbuscules, as specific structures of the fungal component, were present in a low ratio of 0.33%. The colonization degree in this cluster showed a minimum value of 3%. In accordance with the values of the other parameters, the non-mycorrhized areas had the highest value in the entire dendrogram, and the mycorrhizal/non-mycorrhizal area ratio was also the lowest. The largest number of observations, about 27%, was registered in C3. The frequency in this cluster was slightly higher than the average, and the intensity was 30% lower than the frequency. Arbuscules were present in a proportion of more than 1%. About 7% of the dendrogram value could be seen in C4. When compared to the previous cluster, the data in this cluster had a frequency 8% higher than the average and an intensity 4% lower. The values of transfer structures specific to the fungal component were found in these observations at a value of about 5%. Only two data sets constituted C5. These data showed a frequency of about 63%, and the intensity was 30%, half the frequency. Arbuscules were present in a proportion just under 8%. C6 consisted of only one data set. This set of observations had the highest frequency of about 85%, and the intensity was 5% less than the frequency. This cluster had the most arbuscular structures, over 15%, being the only set of observations in the entire dendrogram with high values. Consistent with the values of the other parameters, the non-mycorrhized areas showed low values. The highest value of the mycorrhizal/non-mycorrhizal area ratio was recorded in this cluster. Simultaneously with the values of the other parameters, the colonization degree in these data sets had the highest values. C7 accounted for less than about 8% of the total number of observations. The frequency in this cluster was 10% lower than in the previous cluster. Arbuscules were present in a proportion of less than 1%. Only two data sets constituted C8, and they were characterized by a high frequency and intensity similar to C6. The transfer structures were present in these two data sets at half the value of C6. The last cluster (C9) had a frequency 10% lower than the average and was characterized by only one data set. The intensity did not exceed 19%, and the arbuscules were present in a proportion of over 8%.

### 2.6. Mapping the Native Mycorrhizal Patterns in Festuca rubra

**Figure 5:**
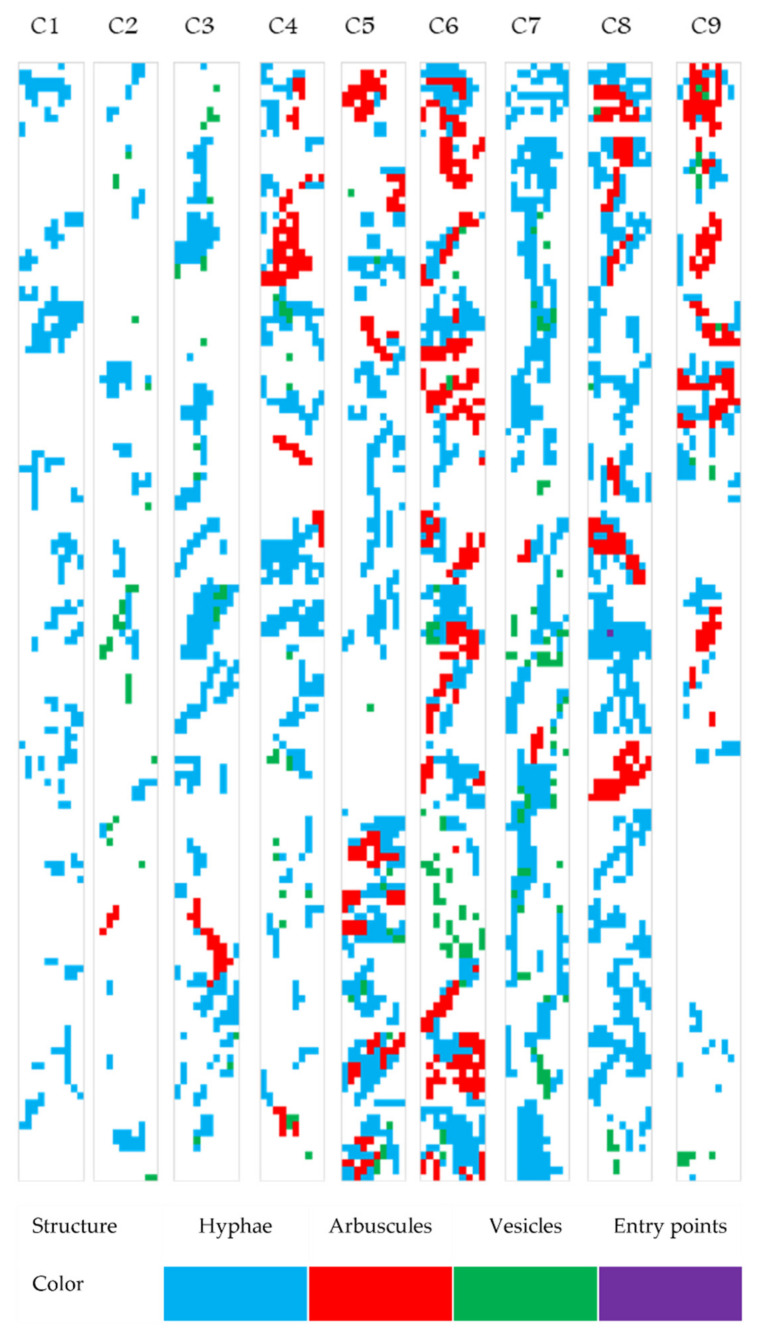
Native mycorrhizal patterns in the roots of *Festuca rubra*. Based on the MycoPatt color code, each structure and the area where it is present are coded by one single color: hyphae (blue), arbuscules (red), vesicles (green), and entry points (purple). C1–C9 represent the specific mycorrhizal map for each cluster described in Figure 4 and Table 2.

The MycoPatt tool, in addition to a multitude of values of mycorrhizal indices, has the option to export colonization maps that show the real position of the fungal component in the root system. A total of nine maps were exported in order to visualize the general trends of root colonization mechanisms in *Festuca rubra* (Figure 5, Table 2). The nine maps were chosen based on the cluster analysis. Each structure/parameter was represented by a specific color. The first map was representative for C1 and contained 25% of the data set. The map (C1) consisted only of a shade of blue, indicating the presence of the fungal component in the root system. These areas alternated with white parts, representing non-colonized areas and demonstrating the rigidity of the species toward the symbiotic process. Map C2 showed arbuscules in small proportions, and the number of hyphae in these observations also decreased. Plant species invest in a symbiont only if it does not have access to enough nutrients. Map C3 represented a combination of maps C1 and C2. Numerous hyphae were present, as well as a number of vesicles and arbuscules. This map represented the mycorrhizal pattern in 27% of the entire registered data set. Due to the high percentage of the appearance of this pattern in recorded observations, we believe that *Festuca rubra* in the grassland ecosystem has a specific malleability for the symbiotic process.

Maps C4 and C5 were similar in terms of the parameters analyzed, but transfer structures began to appear in greater proportions (Figure 5). Given that this type of map was found in less than 10% of the total analyzed data, we believe that *Festuca rubra* does not completely accept the symbiotic process. This allows the fungal component to penetrate the root, to grow intercellularly, but the species shows rigidity for intracellular development toward the formation of arbuscules. On map C6, a peak reached by the arbuscules was visible. In this map, all the three parameters (hyphae, arbuscules, and vesicles) were in almost equal proportions. The map indicates well-established colonization; however, it was present in only 1.6% of the total data. Maps C7 and C8 were similar in terms of structural development of hyphae. Map C7 showed a predominance of vesicles, while map C8 showed a predominance of arbuscules, both with the maintenance of equal hyphae proportion. The last map (C9) was characterized by the presence of fungal components only in the upper part and showed the most non-mycorrhized areas. This pattern of mycorrhizas represents a chaotic colonization in the root cortex. Because it was present only in a proportion of 1.66% of the entire data set, it did not represent a potential mechanism in the final pattern of colonization.

## 3. Discussion

The growth and survival of plants in the extreme conditions of mountain grasslands strongly depend on the efficient use of nutrients and other resources. There are many factors involved in this process. The most important factors that affect practical vegetation are low levels of nutrients (particularly P and N), low temperature, and shortening of the growing season [33]. Therefore, plant species need help in obtaining nutrients in a shorter time, and so symbiosis with mycorrhizal fungi is highly required in such an ecosystem. Mycorrhizal fungi are a group of the most common microorganisms in the soil that influence plant productivity, support above- and below-ground biodiversity [34], and improve the vital functions of the soil that affect plant growth [35] in different ecosystems.

Mycorrhizal symbioses in such ecosystems are a strategy that can limit competition between plant species, with soil fungi directing nutrients to plant species, providing permanence to grassland species. Nevertheless, mycorrhizal fungi can shape the composition of plant communities through the uneven distribution of nutrients among neighboring plants [36]. A large-scale problem in recent years is the appearance and fast development of invasive species in different ecosystems, especially in grasslands. The phenomenon is sustained by the strong associations of these species with soil fungi, which affect or reduce the overall assemblage of native plant biodiversity. One study indicates that the majority (approximately 82%) of the 199 listed invasive plant species are associated with mycorrhizal fungi [37], suggesting that mycorrhizal fungi represent a driver mechanism for plant invasion [38]. In terms of ecosystem conservation, understanding mechanisms underlying plant invasion is vital [39], which raises an important question that should be deeply analyzed in order to evaluate whether invasive species share the same AM species as dominant grasses or whether they are able to harvest different symbionts from the soil microbiome pool. Moreover, studies have revealed that both the composition of root fungal communities and the production of spores can be influenced by host plants [40]. Legumes and grasses, the main categories of grassland taxa, highly depend on the presence of mycorrhizal fungi and N-fixing bacteria [41]. Fungal exudates also induce changes in the structure of bacterial communities. Due to their ubiquity and location in the rhizosphere, mycorrhizal fungi have been named key mutualists with the potential to influence ecosystem processes, such as productivity, carbon and nutrient cycles, water use, and soil structure [42]. These symbionts have a high degree of plasticity in terms of potential interactions with other smaller microorganisms and can have their own endosymbiotic bacteria. In addition, they benefit from the activity of an important group of bacteria that act as helpers for the success of colonization. Bacteria show pre-symbiotic activity and are involved in the promotion of fungal growth and development, followed by an improvement in the connection between mycorrhizas and their hosts [43,44]. Helper bacteria maintain their sustaining activity during the entire life cycle of mycorrhizas, being responsible for stimulating hyphal growth and sporulation.

Each plant taxon has a different survival strategy. Most grasses have of two main options: either invest resources in the formation of lateral roots or associate with a symbiotic partner that will bring a higher intake of nutrients and other benefits. Even if nitrogen is in optimal concentration in the soil, the driving force behind the investment of plants in a fungal symbiont is the presence of phosphates, which are inaccessible to plants. Under oligotrophic conditions, mutual plant–soil reactions can prevent competitive exclusion and favor slow-growing plant species (e.g., germination and increased growth of oligotrophic species by mycorrhizal associations) [45]. Due to the different anatomies and morphologies of plant roots, there are different combinations of fungal species, diversity, and species composition in vegetal communities. The roots can also exert a mutual influence on the associated mycorrhizal fungi [46]. Mycorrhizal fungi can significantly reduce nitrogen leaching losses (up to 70 kg N ha^−1^) and P (up to 150 g P) [47]. Plants that achieve this symbiosis receive nitrogen from inorganic sources and thus can dominate ecosystems that have cycles with high rates of nitrogen mineralization [48]. Up to 25% of plant nitrogen and 80% of plant phosphorus are of mycorrhizal origin [49]. Typically, red fescue has a shallow root system, which is limited to the upper layer, with about 70% of the root biomass concentrated in the upper 20 cm [50]. The roots of *Festuca rubra* growing in mountain grasslands are relatively heavily colonized by arbuscular mycorrhizal fungi [51].

In a grassland ecosystem, most species are perennial and can develop a considerable area of depletion due to prolonged absorption of nutrients from the same soil area [52]. High dominance of some species in plant communities can reduce available resources, promote competitive exclusion, and therefore, reduce plant diversity [53]. The morphology of mycorrhizae varies greatly between species, due to the growth and development of characteristic roots and branching patterns [54]. Gajic’ et al. [55] reported that *Festuca rubra* has a high potential to activate adaptive mechanisms that help withstand stress through increased anthocyanin, phenol, and ascorbic acid biosynthesis and total radical-scavenging activity. Plants that enter this symbiotic partnership enjoy increased protection if the soil is contaminated with heavy metals. Pereira et al. [56] showed that the symbiotic microbiome of *Festuca rubra* is represented by seven taxa belonging to the *Fusarium*, *Diaporthe*, *Helotiales*, *Drechslera*, *Slopeiomyces*, and *Penicillium* genera. Other researchers report that *Festuca rubra* has a high diversity of mycorrhizal fungi and is dominated by the *Glomus*, *Scutellospora*, *Gigaspora*, *Paraglomus*, and *Archaeospora* genera [57]. The structure of the fungal community is driven by plant succession in grasslands, with loose symbiosis in the early stages and a selection of the most suitable species for late succession [58]. Inoculation with mycorrhizal fungi can be an effective phytoremediation solution because it decreases the concentrations of heavy metals that reach the plant organs [59].

This ancient mutualistic symbiosis may be one of the most important but least understood biotic interactions that regulate the plant community structure and dynamics [60]. Grassland managers are interested in understanding the factors that control mycorrhizas, as these symbioses influence the composition of the plant community and the potential protection from soil erosion. Because grasslands are closely related to the forest ecosystem, they are also connected with ectomycorrhizae. The entire forest ecosystem can be considered a biological entity, connected in the underground by a “wood wide web” network formed by ectomycorrhizal fungi, a term coined by the journal *Nature* to highlight the work of Simard et al. [61], and the inter-fungal connections play a vital role in species distribution, community diversity, productivity, and therefore the functioning of ecosystems around the world [62].

This paper may represent a new direction in research on the symbiotic process. The maps extracted from the MycoPatt tool are a complement to genetic studies, as proposed by Šmilauer et al. [63], the analysis of mycorrhizal morphotypes being useful for the assessment of symbiotic mechanisms. The observed general mechanism indicates that each fungal taxon in the root cortex of *Festuca rubra* can produce specific changes in root cells. It is important to be able to observe in which direction the symbiotic process is oriented. The assessment of hyphal compatibility and the hyphal ability to complete the symbiotic process by forming arbuscules is vital to understand the intensity of the exchange of nutrients between partners or whether the fungal component will form more storage structures. The mycorrhizal pattern is a useful method that shows the real position of the symbiont in the roots, as well as the specific structures developed. The maps constructed by the system allow a much easier comparison of patterns. Since it is a digital method based on a large database, subjectivism is eliminated. Most data sets have a frequency slightly above 50%, the overall intensity is 20%, and arbuscules are present in only 1% of the data sets. Based on these values, we believe that *Festuca rubra* has a symbiotic partner acceptance rate of over 50%, indicating that the chosen species is an excellent niche for the fungal component. Another potential application of colonization maps is in monitoring the effects of various chemical elements or heavy metals, especially aluminum, which affects the normal growth of roots, in addition to stopping the obtaining of nutrients.

## 4. Conclusions

The use of colonization frequency classes as a coding variable increases the uniformity of data and improve the analysis of observed differences. Frequency of colonization varied largely between 6% and 93%, sustained by a maximum 40% intensity of colonization. A higher mycorrhizal development potential is visible in the root areas with a greater density of hyphae, with only a 5% maximum for arbuscules development. An increased presence of arbuscules is visible for colonization degree higher than 25%, as a dual fungal strategy for an increased transfer and the continuous development of the hyphal network. Colonization maps shows an irregular development of fungal components, with colonized areas alternating between hyphal development and arbuscules formation. *Festuca rubra* have a native mycorrhizal acceptance rate of over 50%, which makes this species excellent for monitoring the impact of agronomic inputs and applied management. Mycorrhizal maps improve the clarity of symbiosis assessment, allows the analysis of fungal structures based on their real position in root and the colonization strategy.

## 5. Materials and Methods

### 5.1. Experimental Field and Biological Material

The sampling area was a part of a large experimental field (in 4 replications), with organic and mineral fertilizers, established in 2001, in a natural mountain grassland located in the Apuseni Mountains, Romania [64]. In 2020, after 19 years of experimentation, we collected a pool of root samples from the control variant (with no fertilizers applied) of this experiment. Prior to root sampling and extraction, plants were identified in field by two grassland specialists, Dr. Florin Păcurar and Dr. Ioana Vaida, first one being involved in experimental field since its establishment. Soil cores were extracted, and the entire plant was gently removed after this procedure. The entire plant was washed, and roots were detached for the further laboratory steps. The management approach applied for the chosen plot was mowing once per year since establishment. The location was in Ghețari Village, 1130 m above sea level, 46.49064 N–22.81418 E (DD), on a preluvosol (terra rossa) soil type. The soil profile has the following sequence of horizons: At-AO-A/B-Bv-R. The soil volume is small-medium, with a loamy-clayey texture, an apparently low density, and medium total porosity. The soil reaction is acidic, and the humus content is more pronounced in the Ao horizon and lower in the Bv horizon. The available nitrogen supply is medium, with extremely low phosphorus and potassium [65].

*Festuca rubra* is a species of grass known by the common name “red fescue”. At the lower limit, *Festuca rubra* grasslands intertwine with those of *Agrostis capillaris*, descending in some conditions to the nemoral floor at 700–800 m altitude. This species represents a source of food for wildlife and a fodder source, even if the grasslands where it appears dominant are characterized by low productivity. It is a flowering plant (family Poaceae, subfamily Pooideae). The pastoral value of red fescue grasslands is heterogeneous, from mediocre to good, with a production of 5–15 t ha^−1^ of green mass and 0.5–1.5 livestock units ha^−1^ [66]. This species performs under oligotrophic conditions [67]. *Festuca rubra* has a stable symbiotic partnership that is not influenced by the increase in biomass or variations in climatic factors from different years [68]. To identify the native mycorrhizal potential of the soil, a pool of *Festuca rubra* root samples was collected.

### 5.2. Laboratory Techniques for Mycorrhizal Analysis

A large pool of *Festuca rubra* roots was collected for laboratory studies after flowering (15 July 2020), corresponding to the moment of transition from the vegetative to the generative stage. *Festuca rubra* is a perennial plant, with loose to tufted growth [69,70], so plants were harvested from different area of each plot. At harvest, each root sample from each replication was collected in a separate bag. The plant has a shallow root system, with short rhizomes. For mycorrhizal analysis, all roots were cleared and stained before microscopy. The staining method was as described by Stoian and Florian [71], and the following procedure was used: clearing (48 h) in a 10% solution of NaOH and staining with 5% of Pelikan 4001 ink (https://www.pelikan.com/pulse/Pulsar/ro_RO.Store.displayStore.224848./cerneal%C4%83-4001-de-la-pelikan; accessed on 5 November 2021) +5% vinegar solution for another 48 h. This procedure permits good visualization of intra-radical mycorrhizal structures, with slow and delicate entry of solutions in tissues, in the absence of heating.

**Figure 6:**
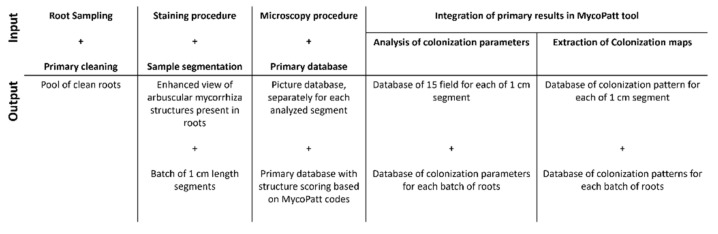
Flowchart of mycorrhizal quantification in the roots of *Festuca rubra*, based on MycoPatt integration.

The quantification of colonization in roots can be expressed only by indicators obtained through microscopy and image captions. The entire process flow is presented in Figure 6. Microscopic evaluation was performed at a magnification of 40× using an Optika microscope, and the mycorrhizal pattern method, proposed by Stoian et al. [32], was used This method evaluates the mycorrhizal colonization in 1 cm root segments. For each segment, 15 microscopic fields were analyzed in order to obtain the entire mycorrhizal colonization image of the segment, and 15 segments were analyzed for each replication, resulting in 60 segments in total. Overall, 900 observations were made, and for each, an image was extracted. All images from one segment were analyzed to assess mycorrhizal structures. The indicators were obtained using the MycoPatt tool, as described by Stoian et al. [32]. The mycorrhizal pattern method, due to it digital characteristics, shows the real position of all fungal structures, along with the automatically calculation of all indicators. It allows the automatic extraction of mycorrhizal maps for each microscopic field from a segment, which shows global mycorrhizal patterns. The entire database resulting from our observations consisted of 900 lines for each of the 7 parameters, further used for colonization analysis: colonization frequency (%), colonization intensity (%), arbuscule and vesicle abundance (%), colonization degree (%), non-mycorrhized areas (%), and the mycorrhizal/non-mycorrhizal area ratio. In addition, each observation represented a map of 10 × 10 squares, which presented an accurate image of colonization in the corresponding root fragment.

### 5.3. Data Analysis

All data were analyzed using R Studio version 1.4.1106 [72] on the R platform [73]. For the entire data set, histograms (the *graphics* package [73]) were used to assess data positioning. We conducted a 2-way histogram analysis for each parameter: The first approach was based on the best-fitted solution, which shows the number of observations for a theoretical mathematic interval; the second approach was based on colonization frequency intervals, which force the positioning of all data for each parameter based on their corresponding frequency class. The colonization frequency was considered both a colonization parameter and a variable restrictor for the rest of the parameters. The *psych* package [74] was used to extract basic statistics, from which means and their standard errors were further used. Scatterplots were used to explore interactions between parameters, and regressions were produced for each interaction using the *stats* package of R [73,75] and verified using the *MASS* package [76,77]. The differences between the means of each parameter, grouped by frequency classes, were analyzed using ANOVA and LSD tests from the *agricolae* package [78], at a significance level of 0.05. The entire set of mycorrhizal parameters was compared using cluster analysis with the *ape* package [79] and with principal component analysis (PCA) and non-metric multidimensional scaling (metaMDS) from the *vegan* package [80].

## Figures and Tables

**Table 1:** Dynamics of colonization parameters in *Festuca rubra*.

	Frequency (%)	Intensity (%)	Arbuscules (%)	Colonization Degree (%)	Non-Mycorrhizal Areas (%)	Mycorrhizal/Non-Mycorrhizal Area Report
M1	6.55 ± 0.38 e	3.59 ± 0.19 e	0.14 ± 0.07 c	0.42 ± 0.03 e	96.41 ± 0.19 a	0.04 ± 0.00 e
M2	30.82 ± 0.49 d	13.17 ± 0.31 d	1.54 ± 0.33 bc	4.18 ± 0.15 d	86.83 ± 0.31 b	0.15 ± 0.00 d
M3	51.27 ± 0.47 c	20.09 ± 0.51 c	1.61 ± 0.40 bc	10.47 ± 0.33 c	79.91 ± 0.51 c	0.26 ± 0.01 c
M4	71.58 ± 0.48 b	27.65 ± 0.67 b	2.76 ± 0.52 b	20.02 ± 0.56 b	72.35 ± 0.67 d	0.41 ± 0.02 b
M5	92.69 ± 0.52 a	38.44 ± 0.86 a	5.21 ± 0.78 a	35.91 ± 0.91 a	61.55 ± 0.86 e	0.68 ± 0.03 a
*F* test	22748	3103	77.97	3038	3103	1433
*p* value	<0.001	<0.001	<0.001	<0.001	<0.001	<0.001

Note: Means ± s.e. followed by different letters present significant differences at *p* < 0.05 according to LSD test. The five classes are based on the frequency of colonization classes: M1, low level (0–20%); M2, low–medium level (21–40%); M3, medium level (41–60%); M4, medium–high level (61–80%); and M5, high level (81–100%).

**Table 2:** Cluster groups defined by assemblage of mycorrhizal strategy based on colonization parameters.

Cluster	Frequency (%)	Intensity (%)	Arbuscules (%)	Colonization Degree (%)	Non-Colonized Areas (%)	Mycorrhizal/Non-Mycorrhizal Area Reports
C1	41.00	14.27	0.00	9.13	85.73	0.20
C2	19.47	8.26	0.33	2.59	91.73	0.09
C3	51.73	20.80	1.40	14.04	79.20	0.30
C4	57.80	23.73	5.27	17.06	76.26	0.37
C5	62.87	29.67	7.87	23.65	70.33	0.52
C6	84.67	39.33	16.53	35.21	60.67	0.77
C7	73.13	33.67	0.73	25.24	66.33	0.53
C8	84.13	38.26	8.00	33.21	33.21	0.67
C9	40.53	18.87	8.53	12.90	81.13	0.31

Note: all values of colonization parameters represent the registered value in the middle point of cluster.

## Data Availability

All data are available by reasonable request from corresponding authors.
